# The Modern Cystoscope; Its Use in Bladder, Ureteral and Kidney Lesions with a Practical Demonstration of the Male Ureter

**Published:** 1898-08

**Authors:** B. O. Coates

**Affiliations:** Cleveland


### Selections and Abstracts.

### THE MODERN CYSTOSCOPE; ITS USE IN BLADDER, URETERAL AND KIDNEY LESIONS WITH A PRACTICAL DEMONSTRATION OF THE MALE URETER.

By B. O. COATES, M.D., Cleveland.

Mr. President and Fellow Practicians: It is not my intention this afternoon to dwell at length on the value of cystoscopic examination in vesical legions, other than to pay a passing glance at some of the more important pathologic changes, with a general outline of the use of the modern cystoscope in vesical, ureteral, and kidney lesions; yet we should not overlook the commoner diseased conditions and the valuable assistance often given in diagnosis by introducing the electric light within the bladder. Since our vision, or what we are able to see, plays such an important part in the recognition of pathologic changes occurring in other parts of the body, it becomes none the less necessary, when advisable, to take an actual view of the interior of the bladder, which will aid us in recognizing the true pathologic changes which occur in the vesical mucous membrane and enable us to watch the progress of the disease and the same under varying forms of treatment.

*"Up to the date of the introduction of the electric illumination of the badder all our knowledge of the obscure diseases of the urinary tract was obtained slowly and with difficulty, for it was acquired either by postmortem or by operative interference." Thus in many instances of early hematuria, with the exception of stone, the exact source of bleeding was somewhat speculative, and indeed, in many cases, quite uncertain. The cystoscope, however, has been of the greatest value in aiding us to locate, frequently to our entire satisfaction, the exact source and cause of bleeding, and to modify in a great measure the significance of the appearance of blood in the urine occurring before, during and after micturition.

A glance at the table which Mr. Fenwick has so nicely arranged will show the difficulty often encountered and the frequent inaccuracy in diagnosing, without a cystoscopic examination, the source and cause of bleeding.

Patients come to us complaining of some urinary trouble, and after a careful study of their symptoms with a thorough examination of the urine, chemical and microscopic, how often we are left quite at sea as to the true nature and cause of their

*E. Hurry Fenwick, F. R. C. S.

ailment, yet we are liable to overlook the fact that our clinical investigation is not complete until we have made an ocular inspection of the interior of the bladder. I consider such inspection quite as essential as the direct examination by illumination of the interior of the ear, larynx, eye, rectum, etc., in their respective lesions.

In certain conditions the diagnostic picture may be strengthened by an inspection of the lining membrane of the bladder by means of the electric light. Such conditions may be new growths, calcific incrustations, small shell calculi left after litholapaxy, small stone and other foreign bodies, simple ulcerations, tubercular and early malignant disease, and such obscure conditions as prolapse of the ureters and vesical mucous membrane, or a small incysted stone held in a pouch or fold of vesical mucous membrane, which almost completely covers it, thus producing symptoms which may entirely mask the true local condition, and yet after careful examination of the interior of the bladder with a steel sound, nothing may be revealed, unless by mere accident.

I take the liberty of quoting one of Mr. Fenwick's cases as an example, and to illustrate the value of the cystoscope in above similar conditions:

"A. P., aged 28, three years ago, being in perfect health and never having had any urinary disease or trouble, jumped off an omnibus while in motion and came with an unexpectedly sudden jerk upon his feet. He immediately felt a severe pain in the crutch, but by putting his finger in the perineum and pressing upwards he gained relief enough to walk home. The pain continued; his doctor sent him to a London hospital, and the surgeon under whose care he was admitted skillfully crushed a calculus weighing 368 grains. Fourteen days after the operation he began to be troubled with renal colic, and since then he has had repeated attacks. The attacks are very short in duration and occur once a day. Pain, not apparently of a severe character, starts in the left kidney and courses along the ureter to the testicle, which is drawn up. On examining the prostate I found the greater part of the interlobar line had gone, and my finger felt as though it would punch through the thin prostatic capsule into the prostatic urethra. I could feel the posterior surface of the pubes easily, and I had no doubt that a calculus had become encysted in the prostate, and the sudden jerk he had sustained in leaping from the omnibus had wrenched it from its bed and had thrown it out into the cavity of the bladder. I could see a deep prostatic pit with the urethroscope and was confirmed in my opinion. I was, however, quite unprepared for what the cystoscopic examination demonstrated.

"In order to ascertain the condition of the left ureter, preparatory to performing nephrolithotomy, I introduced the electric cystoscope and found the entire surface of the bladder n a state of subacute cystitis. After thoroughly washing I saw something which puzzled me greatly. Protruding and retracting from a small hole in the left side of the bladder, above the left ureteral orifice, was a brown spike. I noticed it moved with respiration. It looked like the beak of the sand martin projecting from the hole leading to its nest in the sand-bank or cleft. At first I felt certain it was a stone sticking in the mouth of the ureter, and this inspection explained the presence of renal colic and the absence of bladder symptoms. On putting my finger into the rectum and examining the posterior part of the bladder, I felt, high on the left side, a stony hard body in a sack, and knew at once that the spike I had been looking at was the nose of the encysted stone."

Mr. Fenwick says the curious part of the case is yet to come. From the date of the cystoscopy and the thorough vesical irrigation the patient has been absolutely free from all symptoms of renal colic. "He alleges himself to be cured. He reports himself to me every month or so, and I do not fail to examine this encysted stone. I am inclined to believe its weight had pressed upon and slightly obstructed the left ureteral orifice, the narrowing being increased by the swelling of the subacute cystitis, and that washing and sandal oil, by removing the latter, had removed the partial obstruction. Anyway this encysted stone is symptomless."

I myself saw a patient who had symptoms of stone, and after careful, repeated sounding of the bladder, no stone could be detected, but upon introducing the electric cystoscope a small calculus was seen to be lying imbedded behind the prostate, which was removed a few moments later without difficulty, after which all vesical symptoms subsided.

Prolapse of the ureteral or vesical mucous membrane around the ureteral orifice is, fortunately, not a very common condition, yet when it does occur, and "especially in female children, where it may even protrude through the vulva and tempt the surgeon to ligate it with lethal effect under the belief that it is a vesical growth." A beautiful specimen of such a condition is to be seen in St. Bartholomew's Hospital Museum, No. 2,367, Path. Trans. Vol. 14, page 185.

A very interesting case of the above is that of Caille's, which is described in the "Inter. Jour. Med. Sciences," May, 1888, page 481. A prolapse of the inverted lower third of the right ureter through the urethra of the female child was discovered after a severe fit of crying. This prolapse was apparently owing to the formation of a warty or papillomatous growth, the size of a pea, in the right ureter near its vesical insertion. The protrusion was ligated, and the child died twelve hours afterward. Extensive renal disease was discovered on post-mortem. No cystoscopic examination was made.

That the diagnostic range of the modern cystoscope is not confined to diseases of the bladder, but extends in many cases to those of the kidney with considerable exactness, is an established fact. Not only are we able with its help often to localize the seat of the trouble—*"negative vesical evidence giving a positive diagnosis of renal disease"—but also to distinguish whether there be two "working kidneys," or whether one or both be affected.

It may be said that the cystoscope fails under certain conditions. To some extent this is true, but by keeping in mind three essential points necessary for its employment we may overcome difficulties to a great extent which would otherwise appear to obstruct its use. It can rarely be passed without undue violence—

1. In patients with irregularly enlarged prostates and when the prostatic canal is very devious, as blood becomes smeared on the windows and mixed with the urine in the bladder. An ordinary enlarged prostate offers but little resistance.

2. In stricture of the urethra. This should be dilated or cut. If the meatus is too small to admit of a No. 22 French gauge sound, incise under cocain with a blunt-pointed biscoury.

3. If blood or pus is sufficient in quantity to render the urine non-transparent. This may be removed by washing out the bladder with distilled water, or better, a boracic solution, and refilling with distilled water.

4. In contracted bladders, or in those in which the capacity has been diminished by pressure from without.

5. With spasmodic contraction of the bladder, more especially in tuberculous and other ulcerations. A two per cent. cocain solution usually overcomes this.

6. In certain deformities, such as ankylosed hip, a rickety pelvis or a kyphotic spine. A little adjustment of the head is needed in these cases to obtain a good view.

Passing very briefly over the general assistance often given by the use of this instrument in the diagnosis of cystic lesions, we find another important field for the modern cystoscope, that of catheterizing the ureters. For some time we were content to catheterize the female ureters and that not without attendant and obvious difficulties. These have been largely removed by the modern cystoscope, and a still greater improvement has been made in the instrument of to-day by which we are able to catheterize the male as well as the female ureters. Dr. Brenner, of Vienna, was the first to make a cystoscope for catheterizing the female ureters, which answered the purpose nicely for the time being, but we are indebted to Dr. Nitze, of Berlin, for devising an instrument by which we are able to catheterize the ureter of the male as well as the female. At about the same time, Dr. Casper produced the instrument which

*Willy Meyer, M.D., Genito-urinary Diseases, Vol. I.

I shall have the pleasure of showing you this evening. Neither time nor space will permit me to enter into any detailed discussion as to the merits of the various cystoscopes which have been devised, other than to say that after some experience in the use of the different methods I prefer to use the instrument made by Casper, of Berlin. It is simple in its construction and may be easly rendered aseptic; the curve of the catheter may be regulated at the operator's will to correspond to the line of the ureter; and a desirable feature of this instrument is its size, which is such as will permit of its use without an anesthetic either general or local.

After Dr. Howard Kelly's able address to this society some two years ago, and his excellent method and demonstration of catheterizing the female ureters, I need not remind you of the significance and importance of catheterizing the ureters in the male, as well as the female, especially in some of the obscure kidney and ureteral lesions. As the symptoms are sometimes so varied and the clinical features so misleading, it becomes necessary for the clinician to exhaust every resource at hand in arriving at a diagnosis.

The following cases, some of which I had the pleasure of observing while abroad, will serve to illustrate to some extent and suggest the use of the modern cystoscope, as well as the beneficial results which may be obtained by this, I may say, comparatively simple method of catheterization.

In extreme atrophy or congenital absence of the kidney, always obscure conditions, nothing definite may be obtained from a physical examination, as by palpation, percussion, etc., to make clear the true nature of the condition, yet certain symptoms strongly significant of stone may exist in the side where the kidney is absent and lead us to contemplate operative measures. I saw an extremely interesting example of the latter at St. Peter's Hospital, for stone, London, under the care of Mr. Frayer, to whom I am indebted for a history of the case. J. L. S., aged 62, a bus driver, had stricture for thirteen years which had been cut several times during this period. For years the patient had suffered from pain in the left renal region, associated with hematuria. The pain is severe and subject to severe exacerbations, when the patient describes it as shooting and throbbing, and on these occasions it radiates to the left groin and testicle. No actual sickness occurred during these attacks, but sometimes they were associated with nausea. Usually they last about an hour and a half and occur at varied intervals, frequently twice in twenty-four hours. Increased frequency of micturition (two or three times in an hour) accompanies the attack. For the last two years blood has been seen in the urine, varying in quantity, usually enough to color the urine uniformly, but on one or two occasions it has been in large quantities (the patient speaks of half pints of pure blood). The urine has been frequently thick and foul, and the patient is in the habit of passing bougie and catheter morning and evening to keep his stricture dilated.

Pain.—During and after micturition in the urethra towards the root of the penis.

Stream.—Sometimes fair, usually sprinkling.

Frequency of micturition.—Day, six or seven; night, four or five.

Urine.—Alkaline, Sp. Gy. 1009; albumin, mucus and crystals of triple phosfates.

No cystoscopic examination was made.

From the above clinical symptoms and history of the case Mr. Frayer advised operation and proceeded to perform nephrolithotomy, when, much to his surprise, he found an entire absence of the left kidney. An extraordinary feature of the subsequent history of the case is that all nephritic pain subsided immediately after the operation and the patient had no return of renal symptoms during the four months which had elapsed.

Case II.—I take some pleasure in reporting this rather extraordinary case occurring in Prof. Landau's clinic, Berlin, under the care of A. J. Mainzer: L. A., a married woman, aged 28, had several miscarriages and complained for several years of pain in the right lumbar region, which radiated to the back down the thigh. The pain was of a nephritic character with exacerbations of severe lancinating paroxysms, usually accompanied with nausea, which occurred three or four times in twenty-four hours. She had passed blood at different times. The patient said a diagnosis of stone in the right kidney had been made by two eminent physicians who had advised operative measures. Upon investigation a history of syphilis was found, and the patient had been treated by her family physician for the same. On making a vaginal examination, the ovaries and tubes were found to be normal and the uterus was slightly enlarged but freely movable. Nothing could be elicited by palpation nor percussion, but upon deep pressure acute pain was produced. A cystoscopic examination was made and the bladder was found to be in a healthy condition. On catheterizing the left ureter, nothing abnormal was discovered, but in the other an obstruction some distance from the bladder was found, and by careful manipulation the point of the catheter was made to pass the obstruction. When the urine began to dribble away, and after the removal of a quantity of urine, the patient expressed herself as having immediate relief. It was clearly evident that an obstruction of the ureter existed and it was thought to be a stricture and in all probability of specific origin. Subsequently the patient was treated by regularly catheterizing the offending ureter and the administration of antisyphilitic remedies, after which all nephritic symptoms completely disappeared.

It might be inferred that the symptoms, being of specific origin, would naturally disappear under proper antisyphilitic treatment, but the method employed for immediate relief would seem to justify the use of the ureteral catheter.

Case III.—I am indebted to Dr. W. B. Perry, of Baltimore, for a history of this case, which occurred in Chroback's clinic, Vienna: F. C., aged 30, a married woman with three children, for years had complained of pain in the left lumbar region, radiating to the back and thigh, which was intensified during the monthly periods, and had suffered greatly during pregnancy with frequent micturition, tenesmus and burning of urine. She had been treated for catarrh of the bladder three different times. At the time she was seen all her urinary symptoms were increased and accompanied by a host of nervous and gastric symptoms. On vaginal examination, under an anesthetic, her genital organs were found to be normal. A cystoscopic examination was made and the lining membrane of the bladder looked quite normal, except at the trigone, which was slightly inflamed. The openings of the ureters were normal, but the urine could only be seen flowing from one. The ureters were catheterized and catheters allowed to remain in situ for six hours, during which time the patient was given plenty of water to drink. At the expiration of this period the urine collected from the left kidney was found to be less than one-fourth as much as that collected from the right.

An examination of the urine from the left kidney showed an acid reaction and specific gravity of 1.028. A few epithelial cells, crystals of triple phosfates, were found, but otherwise the urinary constituents were about normal. As regards the urine from the right kidney, aside from the increased quantity nothing abnormal was found.

An exploratory incision was advised and the patient prepared for operation. The lumbar incision was made and a very small kidney was discovered and removed, which measured 1½ inches long by 1 inch in width. The patient made an uneventful recovery without any of the previous nephritic symptoms. A diagnosis had been previously made of atrophy of the kidney, which was verified at the operation.

From a review of what has been said we may learn, by means of the modern cystoscope, the following:

1. The condition of the vesical mucous membrane; the source and frequently the cause of hematuria.

2. The condition of the ureteral lips, and whether urine is being conveyed from both kidneys to the bladder or not. If not, we may learn which of the two is the secreting kidney, and observe the character of the jets of urine propelled from the ureteral cones, whether it be clear, murky or bloody.

3. To collect the urine from each kidney separately, for further examination.

4. To satisfy ourselves as to an existing constriction or obstruction of the ureter which would aid in guiding us as to what course we should pursue.

To decide whether one or both kidneys be affected and to what extent each may be involved, is a question that always confronts the surgeon who contemplates operative measures. Providing no obstruction of the ureter exist whereby the catheter can not be made to pass beyond, I know of no greater aid to, nor better method of determining the character of the excretion, nor for estimating the quality and quantity of the excreting tissue that each individual living kidney possesses, than by using the modern cystoscope with the ureteral catheter adjustment. It would seem that cystoscopy of to-day is no longer an experiment. The surgeon who contemplates operative measures in any doubtful case of vesical or nephritic origin, before deciding what course he should pursue, would do well to exhaust the light that the modern cystoscope may throw upon the apparently dark field, often of obscurity, uncertainty and anxiety.—*Cleveland Journal of Medicine.*

